# The mode of lymphoblastoid cell death in response to gas phase cigarette smoke is dose-dependent

**DOI:** 10.1186/1465-9921-10-82

**Published:** 2009-09-10

**Authors:** Nadia D Sdralia, Alexandra L Patmanidi, Athanassios D Velentzas, Loukas H Margaritis, George E Baltatzis, Dimitris G Hatzinikolaou, Anastasia Stavridou

**Affiliations:** 1 Institute of Biomedical Research and Biotechnology, 55 Solomou Str, Athens 10432, Greece; 2 Faculty of Biology, Department of Cell Biology and Biophysics, University of Athens, Athens 15781, Greece; 3 Faculty of Biology, Department of Botany, University of Athens, Athens 15781, Greece

## Abstract

**Background:**

Cigarette smoke (CS) is the main cause in the development of chronic obstructive pulmonary disease (COPD), the pathogenesis of which is related to an extended inflammatory response. In this study, we investigated the effect of low and high doses of gas phase cigarette smoke (GPS) on cultured lymphocyte progenitor cells, using techniques to assess cell viability and to elucidate whether cells die of apoptosis or necrosis upon exposure to different doses of GPS.

**Methods:**

In our approach we utilised a newly-established system of exposure of cells to GPS that is highly controlled, accurately reproducible and simulates CS dosage and kinetics that take place in the smokers' lung. This system was used to study the mode of cell death upon exposure to GPS in conjunction with a range of techniques widely used for cell death studies such as Annexin V staining, activation of caspase -3, cytoplasmic release of cytochrome C, loss of mitochondrial membrane potential and DNA fragmentation.

**Results:**

Low doses of GPS induced specific apoptotic indexes in CCRF-CEM cells. Specifically, cytochrome C release and cleaved caspase-3 were detected by immunofluorescence, upon treatment with 1-3 puffs GPS. At 4 h post-exposure, caspase-3 activation was observed in western blot analysis, showing a decreasing pattern as GPS doses increased. Concomitant with this behaviour, a dose-dependent change in Δψ_m _depolarization was monitored by flow cytometry 2 h post-exposure, while at 4 h Δψ_m _collapse was observed at the higher doses, indicative of a shift to a necrotic demise. A reduction in DNA fragmentation events produced by 5 puffs GPS as compared to those provoked by 3 puffs GPS, also pointed towards a necrotic response at the higher dose of GPS.

**Conclusion:**

Collectively, our results support that at low doses gas phase cigarette smoke induces apoptosis in cultured T-lymphocytes, whereas at high doses GPS leads to necrotic death, by-passing the characteristic stage of caspase-3 activation and, thus, the apoptotic route.

## Background

Tobacco smoke contains more than 4000 compounds [1,2] that have been shown to cause carcinogenesis and other serious lung diseases, such as chronic obstructive pulmonary disease (COPD) [3-6]. Cigarette smoke (CS) consists of the gaseous phase (GPS) and the particulate matter (tar) [7]. Although the carcinogenic properties of chemicals in tar are well known [8], more recent studies have emerged demonstrating major cytotoxic effects on pulmonary and immune cells attributed to the gaseous phase [7,9-11]. The effect of these compounds can be both direct on the most critical line of defence of the airway epithelium [7,12,13] and indirect evoking immune responses, which in turn have a deleterious effect on lung structure [13,14]. In the case of COPD, the progressive destruction of pulmonary tissue has been attributed to inflammation, oxidative stress and proteolysis, the underlying death mechanism of which is still a matter under debate. However, several studies have clearly shown that metabolically-activated or direct action genotoxic components and inhibitors of DNA repair in GPS may contribute to DNA damage and to smoking-related diseases of the upper aero-digestive tract [15].

In the past decade, a number of studies were carried out in order to characterise the mode of death of cells challenged with different doses of cigarette smoke [16-19]. Taking this into consideration, there has been increasingly intense interest in the effects of GPS. A common denominator in many of these *in vitro *studies has been an overwhelming system for CS administration. The practice of cigarette smoke extract or condensate (CSE or CSC) assumes the application of a large quantity of toxic substances on cell cultures, since the toxic load of a whole cigarette is withheld within a relatively small volume of diluents [20-22]. This locally creates a direct and appropriate critical mass of toxic substances, so that the defence mechanisms of the cells are promptly depleted. Such cumulative condition with large quantities of toxic/carcinogenic substances in the cell culture could occur only with exceptional difficulty during normal smoking.

Various studies present conflicting evidence as to whether cells exposed to tobacco smoke die of apoptosis or due to necrosis, or both [16-20,22]. Given that the approach of CSE or CSC administration relates to overdosing cultured cells with CS constituents, then it is not surprising that many of these studies support the idea of necrotic death. Our approach is unique as we employed a method [11,23] for highly controlled and accurately reproducible cell exposure to gas phase CS that closely resembles the dosage and gas kinetics of CS in the smokers' lung, in conjunction with standard techniques to evaluate and quantify the mode of cellular death. In our study, we utilised a well-established lymphoblast cell line to examine CS toxicity *in vitro*. The lymphocyte cell system has previously been used in cell death research and is now considered a model system for similar studies [24-26]. In our experiments, the use of the CCRF-CEM cell line served an additional purpose: T cells are widely recruited in the sites of lung inflammation attributed to CS [27]; however, their precise function and involvement in lung tissue destruction remain to be elucidated. It is therefore of paramount importance to study the fate of T cells in response to various doses of tobacco smoke *in vitro*. Our results clearly demonstrate that the effects of CS administration are both dose- and time-dependent and that apoptosis is an active process triggered by tobacco smoke constituents at low toxicity. Necrosis, on the other hand, is a predominant phenomenon in cultures exposed to high toxicity GPS.

## Methods

### Cell culture

The human T-lymphoblastoid cell line CCRF-CEM (ATCC cat. No. CCL-119) was maintained in RPMI 1640 medium (Biochrom, Berlin, Germany) supplemented with 10% fetal bovine serum (FBS), L-glutamine (2 mM) and penicillin/streptomycin (100 U/ml). Cultures were grown in suspension in a 37°C/5% CO_2 _humidified incubator. Prior to experiments, cells were counted on a Neubauer Haemocytometer and cell viability was assessed with 0.5% Trypan Blue staining. For experimental purposes, cells were transferred to 6-well or 96-well tissue culture plates (Greiner Bio-One, Austria) at a density of 1 × 10^6 ^cells/ml, unless otherwise stated.

### Cell exposure to Gas Phase Smoke (GPS)

Kentucky 1R3F research-reference filter cigarettes (The Tobacco Research Institute, University of Kentucky, Lexington, KY) were used throughout this study. Prior to use, cigarettes were conditioned for at least 48 h (up to 6 days), in a controlled environment chamber (Environ-Cab, Lab-Line Instruments Inc., IL, USA) at 22 ± 0.5°C temperature and 60 ± 1% humidity. Smoke was generated with a mechanical smoking machine (SM410, Cerulean, UK) according to ISO rules (2 seconds puff duration, 35 ml puff, bell shape puff profile, 1 minute puff cycle). In order to remove the particulate matter and obtain gas phase smoke (GPS), the cigarette smoke was passed through Cambridge filters rated to withhold 99.9% of all particles > 0.01 μm in diameter.

The second puff of a single 1R3F cigarette was used to generate each puff of GPS. The GPS was pumped directly into a gas-tight volumetric exposure chamber containing the cells in the lid-less multi-well format plates. Following GPS exposure, the cells were returned to the 37°C/5% CO_2 _incubator for the specified incubation time.

### Cytotoxicity Assay

Cytotoxicity was assessed using the LDH assay (Roche Applied Science, Indianapolis, IN, USA), according to the manufacturer's instructions. Briefly, 2 × 10^4 ^cells per well were seeded in four flat-bottomed 96-well plates. The cells were treated with the required GPS dose (1, 3 or 5 puffs), whereas a plate was left untreated (control). Five replicates were included for each sample.

All cells were washed in 1% FBS medium and were finally resuspended in 1% FBS medium for assaying purposes. In the control plate, one row of cells was resuspended in 1% Triton buffer (1% Triton-X-100 in 1% FBS medium) and was incubated at 37°C for the maximum time allowed (24 hours) to assay for the maximum amount of LDH released from the cells (high control). Control cells that were assayed for LDH immediately after seeding provided the low control to determine basal levels of LDH release in the cell population. LDH was also assayed for in the 1% FBS medium to correct for LDH background in serum. Experimental samples were assayed at 1, 4 and 24 hours post-exposure to GPS. Like control cells, the treated samples were washed and resuspended in 1% FBS medium prior to assaying.

Following incubation, the supernatants of all samples were collected and spun to rid of cell remnants. The cleared supernatants were mixed 1:1 with the dye/catalyst mix, as per the manufacturer's protocol. The amount of LDH was measured using a TECAN spectrofluorimeter at 430 nm, using a 620 nm reference filter. Percent (%) cytotoxicity was calculated using the average of the 5 replicates and the formula provided by the manufacturer.

### Annexin V-Propidium Iodide assay

To determine the percentage of apoptotic cells and differentiate these from necrotic populations, an Annexin V-fluorescein isothiocyanate (FITC)/Propidium Iodide (PI) detection kit (556547, BD Biosciences, UK) was used.

CCRF-CEM cells were exposed to 1, 3 or 5 puffs of GPS and were subsequently incubated for 2 hours. At the end of the incubation period, cells were collected, washed in cold PBS and stained with Annexin V FITC/PI, according to the manufacturer's instructions. Untreated cells were also stained, in order to determine the spontaneous apoptotic index of the cellular population. A 2 μM staurosporine (S4400, Sigma-Aldrich) (STS)-treated cell population, which was also harvested at 2 hours, was included as a positive control for apoptosis. Vehicle control was also included.

The cell suspensions were immediately analyzed using a FACSCalibur flow cytometer (BD Biosciences, UK), equipped with a 488 nm argon laser and the appropriate filter sets. Green fluorescence for FITC was collected using a 530/30 bandpass filter and red fluorescence for PI using a 585/42 bandpass filter. For each sample, ten thousands events were acquired and statistically analysed using CellQuest software version 7.5.3 (BD Biosciences, UK).

### FACS analysis of mitochondrial membrane potential

For analysis of the mitochondrial inner membrane potential (Δψ_m_) in whole cells, the membrane-permeable lipophilic cationic fluorochrome JC-1 was utilised (Mitoscreen kit, BD Biosciences, UK). In live cells, JC-1 exhibits potential-dependent accumulation in mitochondria forming J-aggregates. These aggregates can be detected within the red fluorescence spectrum (~590 nm), in contrast to the green fluorescence (~529 nm) emitted by JC-1 monomers. An increase in green fluorescence indicates depolarization of the mitochondrial membrane potential.

Briefly, CCRF-CEM cells, treated with GPS or STS as previously described, were collected by centrifugation (400 g). The cells were resuspended at 1 × 10^6^/ml in pre-warmed JC-1 working buffer containing 2 μM JC-1 and incubated for 15 min in a 37°C/5% CO_2 _incubator. Subsequently, the cells were washed in assay buffer and directly analyzed in a FACSCalibur flow cytometer using the appropriate filter settings. Red and green populations were gated for quantification analysis using CellQuest software. Ten thousands events were acquired for each sample.

### Western Blot Analysis

Whole cell lysates were prepared in RIPA buffer (50 mM Tris-HCl pH 8, 150 mM NaCl, 1 mM EDTA, 1 mM DTT, 0.5% NP-40) on ice, including a mix of protease inhibitors (P8340, Sigma-Aldrich). For cytoplasmic extracts, the lysates were centrifuged at 12,000 g for 15 min, at 4°C. Protein concentration was measured using the Bradford assay (B6916, Sigma-Aldrich) and approximately 40 μg from each sample were boiled in Laemmli buffer (50 mM Tris-HCL pH 6.8, 2% SDS, 1,25% β-MSH, 5% glycerol, 0.0125% bromophenol blue). Proteins were analysed on 11% SDS-PAGE followed by transfer onto nitrocellulose membrane. Active caspase-3 was detected using a commercially available antiserum (1:100; AB3623, Chemicon Millipore-MA, USA) and labelled with a HRP-conjugated secondary antibody (AV132P, Chemicon Millipore). For loading control, a monoclonal anti-a tubulin antiserum (MCA78G, ABD Serotec) was used (1:500) to identify cellular tubulin, together with an anti-rat HRP-conjugate (A9037, Sigma-Aldrich). The blots were developed using Amersham ECL Kit (GE Healthcare, UK).

### Confocal microscopy

Control cells or cells exposed to 1, 3 or 5 puffs GPS and harvested at 1, 4 or 24 hours post-exposure were fixed in 4% paraformaldehyde/PBS, pH 6.9. The cells were permeabilised with 0.1% Triton-X-100 and non-specific sites were blocked with 1% BSA in PBS. As CCRF CEM were grown in suspension, prior to staining, cells were attached onto microscope slides using Shandon Cytospin Cytocentrifuge (Thermo Scientific, MA, USA).

Active caspase-3 staining was performed using a commercial antiserum (1:150; 9661; Cell Signaling, USA) and anti-rabbit FITC antibody (1:80; F0382, Sigma-Aldrich). Cytochrome C was identified using a monoclonal antibody (1:150; 13561; Santa Cruz Biotechnology) and anti-mouse Alexa 488 conjugate (1:100; A11029; Molecular Probes). Where necessary, nuclear counterstaining with 1 μg/ml propidium iodide (PI) was included. Apoptosis was induced using 2 μM staurosporine (positive control). Secondary antibody negative controls were also included.

The samples were visualised using a Nikon C1 Digital Eclipse Confocal Microscope system, equipped with a 488 nm Argon and a 543 Helium Neon laser through an oil immersion ×60/1.4 objective.

### Detection of DNA fragmentation by flow cytometry

DNA fragmentation was assessed in smoke-treated cells and compared to healthy cells, as well as staurosporine-treated apoptotic cells using the Apo-BrdU Kit (556405, BD Biosciences, UK).

Approximately 2 × 10^6 ^cells were collected and briefly fixed in 1% paraformaldehyde/PBS, pH 6.9, followed by overnight fixation in 70% ethanol at -20°C. TdT-catalysed end-labelling of fragmented DNA with bromolated deoxyuridine triphosphates (Br-dUTP) was carried out at 37°C. End-labelled DNA was probed with anti-BrdU monoclonal antiserum provided in the kit. All cells were counterstained with a 5 μg/ml PI/200 μg/ml RNAse A solution.

All samples were analysed using a FACSCalibur cytometer and the appropriate green/red filter settings. Ten thousand events from each sample were analysed.

### Statistical analysis

All data presentations (graphs etc.) and corresponding statistical analysis was performed using SigmaPlot and SigmaStat software packages (SPSS Inc.). All data in graphs are expressed as mean values ± SD. For one way ANOVA analysis a *P *< 0.05 was considered significant.

## Results

The results described are representative of three or more independent experiments.

### Cytotoxicity measurements

**Figure 1:**
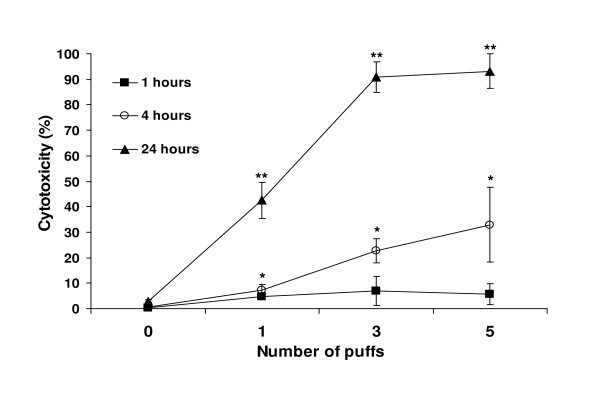
**Cytotoxicity of GPS-exposed cells increases in a dose- and time-dependent manner**. Cytotoxicity was measured in terms of LDH release in the culture medium from cells exposed to 1, 3 or 5 puffs at 1, 4 and 24 hours post-exposure. The graph incorporates mean values ± SD of data derived from one representative experiment of three independent series performed in quintuplets. * *P *< 0.05 compared with control, ** *P *< 0.0001 compared with control (one-way ANOVA).

The cytotoxicity of gas phase cigarette smoke (GPS) on cultured lymphocytes was assessed using a kit to measure lactate dehydrogenase (LDH) release from compromised cell membranes (Figure 1). The amount of LDH released from cells exposed to the different doses of GPS (1, 3 or 5 puffs) at 1, 4 or 24 hours post-exposure was directly compared to the amount of enzyme from untreated cells (low control-basal levels of LDH in the cell culture) and cells treated for the lengthiest part of the experiment (24 hours) with 1% Triton-X buffer (1% Triton-X in 1% FBS medium) (high control-maximum LDH release). The mean of measurements for the spontaneous LDH activity in the culture media due to the presence of serum was subtracted from all experimental values.

The measurements taken at 1 hour post-exposure were not significantly different (*p *≥ 0.47) among all three GPS doses. The average percentage of cytotoxicity at that exposure time was about 5.8% (± 0.8%). At 4 hours post-exposure, the percentages were markedly different, demonstrating a dose- and time-dependent increase in cytotoxicity. Exposure to 1 puff GPS resulted in cytotoxic death of approximately 7.15 ± 2.42% of the cells. The percentages were more than three-fold (22.76 ± 4.65%) and quadra-fold (32.92 ± 14.77%) higher for cells treated with 3 and 5 puffs GPS, respectively. It has to be noted, that the high standard deviation of the 5-puff data at 4 hours exposure did not allow for a statistically significant differentiation between the 3 and 5 puffs cytotoxicities, as determined by one-way ANOVA analysis (*p *< 0.22) although both 3 and 5 puff data were significantly different than the data for 1 puff (*p *< 0.05). At 24 hours post-exposure, LDH release from cells revealed the same cytotoxicity pattern. Cells treated with 1 puff GPS reached 42.46 ± 7.07% cytotoxicity, which was significantly lower than the percentages recorded for the cells treated with either 3 or 5 puffs (90.89 ± 5.98% and 93.49 ± 6.75%, respectively). As with 4 hours post exposure, at 24 hours the percentages of cytotoxicity of the cells treated with the higher doses (3 and 5 puffs) were not significantly different (*p *< 0.62).

### FACS analysis of Annexin V/PI-stained cells

**Figure 2:**
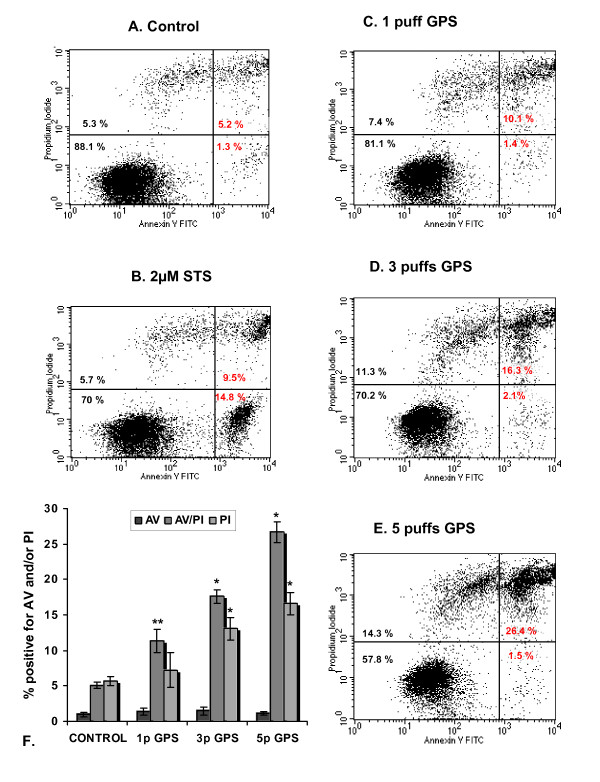
**GPS induces apoptotic and necrotic cell death in CCRF-CEM cells**. CCRF-CEM cells were exposed to various doses of GPS (1, 3 or 5 puffs) and stained with Annexin V/PI, followed by flow cytometry analysis 2hr post-exposure. Panels A-E depict representative data. Lower left quadrants represent unstained cells and the upper left quadrants include PI-positive cells. The lower right quadrants encompass Annexin V-only positive and the upper right contain the Annexin V-FITC/PI-stained cells. **A) **control cells (untreated), **B) **2 μM STS, **C) **1 puff GPS, **D) **3 puffs GPS, **E) **5 puffs GPS. **F) **The plot represents mean values (± SD) of events for stained cells obtained from three independent experiments. * *P *< 0.0001 compared with control, ** *P *< 0.001 compared with control (one-way ANOVA).

To determine the mode of cell death upon GPS treatment, cells were stained with AnnexinV/PI and analysed by flow cytometry, 2 h post-exposure. As seen in Figure 2, the percentage of the Annexin V-stained populations (early apoptotic cells - lower right quadrants) in the control group and all groups of GPS-treated cells were not significantly different (one-way ANOVA, *p *< 0.43).

A clear dose-dependent increase of cells stained with both Annexin V and PI (late apoptotic cells - upper right quadrants) was observed (one-way ANOVA, *p *< 0.001). The percentage of double-stained cells treated with 1 puff was 11.37 ± 1.45% compared to 5.10 ± 0.41% for the untreated cells, representing an almost 2-fold increase. At higher toxicity conditions, there was a steady increase in the AnnexinV/PI-positive cell numbers, with the corresponding population reaching 17.62 ± 0.82% at 3 puffs and 26.66 ± 1.66% at 5 puffs. A similar pattern was observed in the PI-stained cell population (necrotic cells; upper left quadrants) as judged by one way ANOVA (*p *< 0.0001), with the exception of the 1 puff treated cells that showed no statistically significant differences compared to the control (untreated) cells group (*p *< 0.33).

### Analysis of the mitochondrial membrane potential

The dissipation of the mitochondrial inner membrane potential (Δψ_m_) is considered as an early sign of apoptosis, preceding phosphatidylserine exposure on the outer plasma membrane [28]. In necrotic cells, Δψ_m _and mitochondrial integrity are irreversibly compromised. In order to typify the mode of GPS- induced cell death, we examined the status of the mitochondrial membrane potential, Δψ_m_, from cells treated with different doses of GPS, using the marker JC-1.

**Figure 3:**
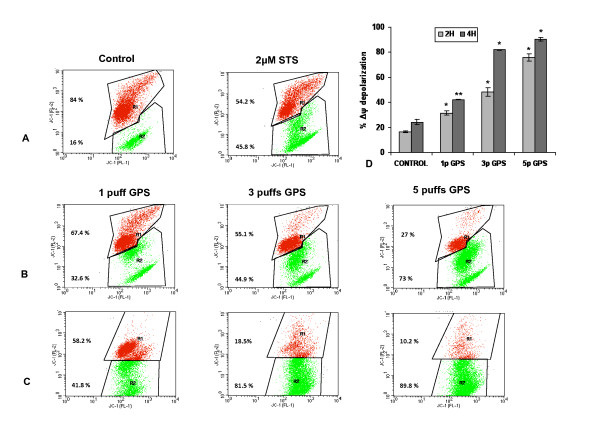
**Loss of mitochondrial membrane potential is both GPS dose-dependent and time-dependent**. Δψ_m _depolarization monitored by FACS analysis of JC-1 mitochondrial potential marker staining 2 h post-exposure (panels A and B), and 4 h post-exposure (panel C). In panels A-C representative dot plots from a single analysis are shown. Gated region R1 (red) includes cells with intact mitochondrial membranes and gated region R2 (green) depicts cells with loss of Δψ_m_. **A) **Control (untreated) cells and cells treated with 2 μM staurosporine (STS; positive control) for 2 h. **B) **1-5 puffs GPS-treated samples analyzed 2 h post-exposure and **C) **4 h post-exposure. **D) **Graphic representation of mean values for R2 region data (cells with Δψ_m _collapse) ± SD. Asterisks above bars denote *p *values for one way ANOVA analysis: * *P *< 0.0001 compared with control, ** *P *< 0.001 compared with control. Analyzed data derived from 4 and 3 independent experiments performed for the 2 h and 4 h time points, respectively.

The status of the mitochondrial membrane potential was examined initially at 2 hours post-exposure (Figure 3A-B). Following exposure to 1 puff GPS, the cell population with disrupted Δψ_m _(green), was almost double (31.62 ± 1.74%) compared to control cells (16.64 ± 0.52%). At higher doses, green fluorescence increased remarkably, reaching 48.27 ± 3.18% for cells treated with 3 puffs and 75.90 ± 3.07% for cells exposed to 5 puffs. The results were more prominent at 4 h post exposure (Figure 3C), especially for cells treated with the higher doses. Δψ_m _depolarization ascended to 81.90 ± 0.40% for the cell sample treated with 3 puffs and to 90.24 ± 1.33% for cells exposed to 5 puffs GPS. Cells exposed to 1 puff, at 4 hours post-exposure did not exhibit such a dramatic increase in the percentage of the population (42.06 ± 2.03%) with disrupted Δψ_m _when compared to the equivalent at 2 hours post-exposure. One way ANOVA analysis among groups of data for untreated and GPS-treated samples showed high statistical significance (*p *< 0.001 or *p *< 0.0001) in all cases, both for 2 hours and 4 hours examined samples, accentuating the observed dose-dependent effect of GPS on the depolarization of the mitochondrial potential in treated cells.

### Confocal microscopy of cytochrome C and active caspase-3

Confocal laser scanning microscopy was utilised to visualise two events that are characteristic in the classical apoptotic process: the cytoplasmic release of cytochrome C from compromised mitochondria and the downstream activation of caspase-3.

**Figure 4:**
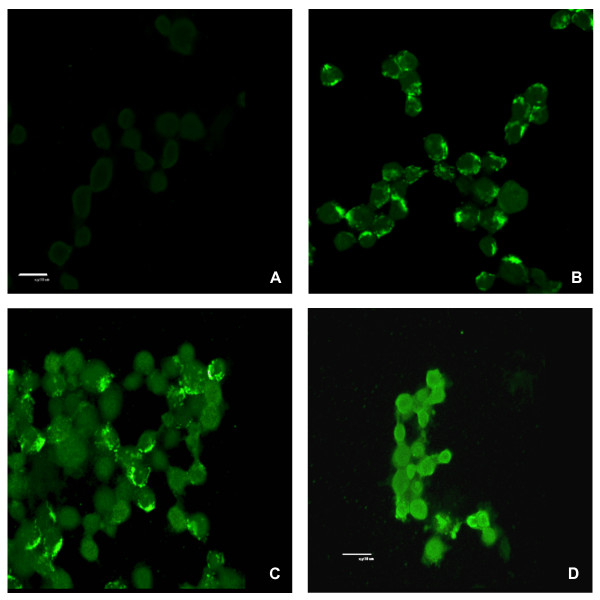
**GPS treatment results in the cytoplasmic release of cytochrome C**. Confocal microscopy of cytochrome C localisation in untreated and GPS-exposed (3 puffs) CCRF-CEM cells: **A) **secondary antibody control, **B) **untreated cells, **C) **3 puff GPS-treated cells at 4 hr post-exposure and **D) **24 hr post-exposure. Scale bar = 10 μm.

Cells treated with 1, 3 or 5 puffs were harvested and fixed in 4% paraformaldehyde/PBS, pH 6.9 at 1, 4 or 24 hours post-exposure. Staining of untreated cells for cytochrome C (Figure 4B) showed bright fluorescence, which was localised in a distinct pattern in the perinuclear area. Cells treated with 1 puff, exhibited diffuse cytoplasmic staining for cytochrome C from 4 hours post-exposure (data not shown). Cells treated with 3 puffs GPS showed a widespread cytoplasmic staining pattern, resembling that observed in the staurosporine control, which increased in a time-dependent manner (Figure 4C-D). At 5 puffs GPS, cytoplasmic staining appeared as early as 1 hour post-exposure, and by 24 hours almost every cell was shrunk and exhibited a diffuse, yet fading pattern of fluorescence (data not shown).

**Figure 5:**
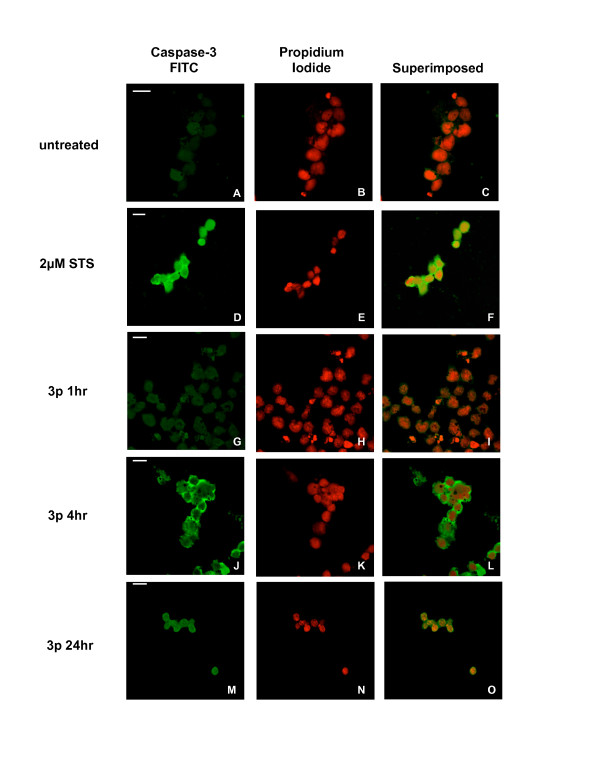
**Activation of caspase-3 in response of cell exposure to GPS**. Confocal microscopic examination of CCRF-CEM cells exposed to 3 puffs GPS for the activation of caspase-3. First row: FITC-staining (green), second row: PI counterstain (red), third row: superimposed FITC/PI images. **A-C) **untreated cells, **D-F) **cells treated with 2 μM staurosporine, **G-I) **cells harvested at 4 hr post-exposure, **J**-**L) **cells harvested at 24 hr post-exposure. Scale bar = 10 μm.

Cells treated with 1 or 3 puffs GPS and stained for active caspase-3 exhibited a gradual increase in the occurrence of FITC-positive cells over time during the acute phase (1 and 4 hours post-exposure) (Figure 5, selected data shown). At 4 hours post-exposure (Figure 5, panels 5J-5L), the detected fluorescence was similar to the stauroporine control (panels 5D-5F) with some blebbing apparent. By 24 hours, the cells looked markedly shrunk and staining was non-specific (panels J-L). Moreover, 5 puff GPS treatment resulted in extremely limited signal at 4 hours and non-specific signal at 24 hours (Panels 5M)-5O).

### Immunoblot analysis of active caspase-3

**Figure 6:**
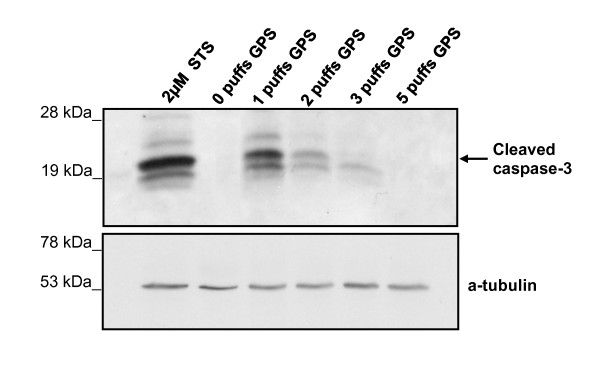
**GPS causes a dose-dependent change in active caspase-3 protein levels**. Western blot showing cleaved caspase-3 in CCRF-CEM cells exposed to 0, 1, 2, 3, or 5 puffs GPS and analyzed 4 hr later. Lane 1) 2 μM STS-treated cells, lanes 2-6: 0-5 puffs GPS respectively. Active caspase-3, showed by an arrow, was recognized by a specific polyclonal antibody (AB3623, Chemicon), that reacts with the 17 kDa cleaved form of the enzyme. Equal loading was verified using α-tubulin as sample internal protein control. The blot is representative of three independent experiments.

Caspase-3 activation was detected in Western blots probed with a specific polyclonal antibody that recognized the 17 kDa cleaved form of caspase-3 (Figure 6). CCRF-CEM cells were treated with 1, 2, 3 or 5 puffs of GPS and samples were harvested 30 min, 1, 2, 4 and 24 hours post-exposure. In apoptosis-positive control cells, apoptosis and caspase-3 activation were induced for 2 hours with 2 μM staurosporine.

In immunoblots, optimal signal for active caspase-3 was detected 4 hours post-exposure in the samples exposed to 1, 2 and 3 puffs, whereas in samples treated with 5 puffs, caspase-3 cleavage was undetected at all time-points examined (data not shown). At 4 hours post-exposure (Figure 5 panels J-L), caspase-3 activation was most prominent in the sample treated with 1 puff (Figure 6). Detection of the cleaved caspase-3 gradually decreased in the rest of the samples, as the number of puffs increased. Active caspase-3 was absent from the sample exposed to 5 puffs. Equal loading was verified by probing the samples analysed with an anti-α-tubulin antiserum.

### DNA fragmentation analysis by flow cytometry

**Figure 7:**
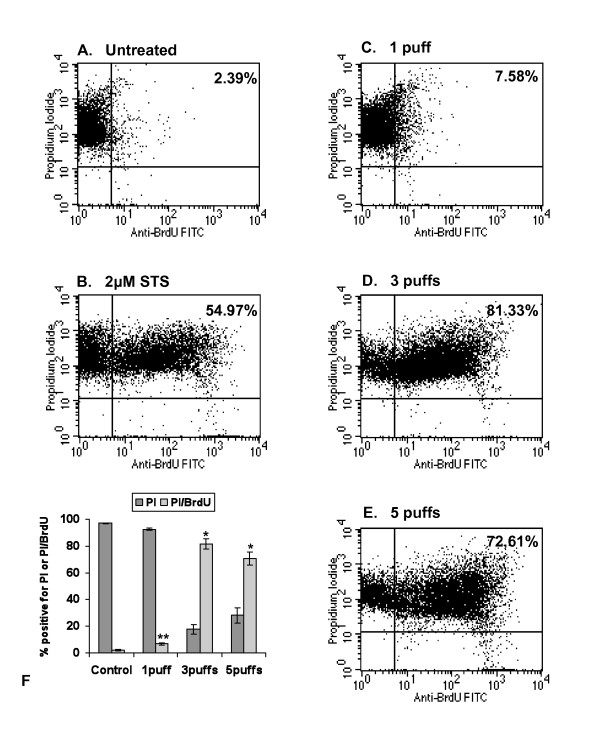
**FACS analysis of DNA fragmentation following exposure to GPS**. Cells exposed to 1, 3 or 5 puffs GPS were harvested 24 hours post-exposure and analysed for DNA fragmentation using BrdU labelling and PI counterstaining. **A) **untreated cells, **B) **2 μM STS, **C) **1 puff, **D) **3 puffs, **E) **5 puffs. **F) **Bar chart showing the cumulative results for PI-only stained cells and PI/BrdU stained cells, derived from 3 different sets of experiments (mean values ± SD). * *P *< 0.0001 compared with control, ** *P *< 0.001 compared with control (one-way ANOVA).

DNA fragmentation of cells treated with GPS was assessed quantitatively using DNA end-labelling (TUNEL) and flow cytometry (Figure 7). Cell populations exposed to 1, 3 or 5 puffs of GPS were treated with BrdU and DNA nicks were identified with an anti-BrdU monoclonal antiserum.

The untreated cells (Figure 7A) demonstrated basal levels (2.20 ± 0.48%) of DNA fragmentation (BrdU/PI-positive cells; upper right quadrant), with the majority of the population (97.09 ± 0.25%) located at the upper left quadrant (propidium iodide staining). The positive control was indicative of the DNA fragmentation occurring upon a two-hour induction of apoptosis with 2 μM staurosporine (Figure 7B). When treated with 1 puff GPS, there was almost a three-fold increase in the cell population with fragmented DNA (6.95 ± 0.65%) (Figure 7C), when compared to the negative control (one-way ANOVA, *p *< 0.0011). The cells treated with 3 puffs showed a maximum population stained for BrdU incorporation (81.77 ± 3.11%) (Figure 7D). At 5 puffs (Figure 7E), the BrdU/PI-positive population revealed a statistically significant decrease (70.78 ± 3.99%, *p *< 0.037) when compared to cells exposed to 3 puffs.

## Discussion

In smokers' lungs, circulating lymphocytes are exposed to cigarette smoke through the wall of the capillary vessels on the surface of alveoli. T cells are one of the major groups of immune cells activated and recruited at the sites of lung lesions caused by CS inhalation [3,27,29]. In our study, we focused on the immediate effects of the gaseous phase of CS (GPS) on T lymphocytes, a cell group of the immune system, which has received little emphasis in the past. The objective was to determine the mode of lymphocyte cell death upon exposure to the gaseous phase of cigarette smoke (GPS) *in vitro*. For this purpose, we utilised a well-established T lymphoblast cell line to examine the effects of GPS.

Our results demonstrated that the mode of cell death was dose-dependent. We examined early and late events in the apoptotic pathway using cells exposed to low (1-2 puffs) and higher (3 or 5 puffs) doses of the gaseous phase. Experiments pertaining to the cytoplasmic release of cytochrome C and the subsequent activation of caspase-3, collectively pointed towards the activation of the caspase-3 dependent apoptotic pathway in a dose-dependent, as well as time-dependent manner. Furthermore, our results from the quantitative evaluation of the mitochondrial inner membrane potential and the late event of DNA fragmentation further supported a dose- and time-dependent change in the mode of cell death, albeit both DNA fragmentation [30] and mitochondrial inner membrane depolarization can occur both in apoptotic and necrotic cells [18].

Annexin V detection of phosphatidylserines on the outer plasma membrane indicated a dose-dependent increase in cell death, although it did not provide a solid basis for discrimination between apoptotic and necrotic death. The presence of Annexin V-positive cells at the higher doses of GPS cannot rule out a caspase-independent death. The apoptosis-specific markers cytochrome C and active caspase 3 prevailed at the low dose (1 puff) and partly at some of the higher doses (3 puffs) up to 4 hours post-exposure. Therefore, our findings are in agreement with previous work that supported a caspase 3-dependent apoptotic death [31,32]. In our system, we observed a dose-dependent decrease in caspase-3 activation, as GPS-doses increased. A switch from apoptosis to necrosis was evident in samples examined at a later time-point (24 h), mainly in cells treated with 3 puffs. The use of the higher dose (5 puffs) resulted mainly in necrotic death, as caspase-3 activation was undetectable. This was further supported by examination of the mitochondrial membrane potential (Δψ_m_) of cell treated with low or high doses of GPS. At low toxicity, Δψ_m _was disturbed enough so that caspase-dependent apoptosis would follow. When exposed to high toxicity, the majority of the cell populations exhibited great loss of Δψ_m_, thus becoming deprived of mitochondrial ATP production, which is required for an apoptotic response together with cytosolic ATP [33]. Similarly, the results from DNA fragmentation point towards a dose-dependent transition from apoptosis to necrosis. This was most evident in the cell populations examined following exposure to 3 or 5 puffs. Although the cells exposed to the 3 puffs showed a maximum of BrdU/PI-positives, at 5 puffs the equivalent population was a lot less. Perhaps, the toxic shock that lead to the depletion of intracellular ATP resulted in the inhibition of endonucleases, which require ATP to be active [30]. Yet, necrosis following caspase-independent apoptosis cannot be ruled out.

Earlier studies supported that treatment with cigarette smoke condensate or extract (CSC or CSE) resulted in apoptosis in a range of cell lines, such as A549 alveolar epithelial cells [34,35], HFL-1 lung fibroblasts [22], human aortic endothelial cells [31], human umbilical vein endothelial cells [32] and alveolar macrophages [36]. Some cases showed a dose-dependent increase of activated caspase-3 [31,32]. There has also been evidence for caspase-independent apoptosis in CSE-treated cells, as shown with the use of caspase inhibitors [34,36].

Other groups concluded that necrosis was the only outcome following CSE treatment of A549, Jurkat and human umbilical vein cells [37], or human primary and BEAS-2B cells [18,20]. Finally, other research supported that CSE induced both apoptosis and necrosis in a dose-dependent manner in A549 [34], HFL-1 [38], U937 human premonocytic [39] and BEAS-2B cells [40].

Most of the times, the application of CSC or CSE on cultured cells assumes the concentration of the toxic components of one full-flavoured cigarette in a small volume of saline buffer or growth medium. The practice of CSC or CSE results in an overwhelming toxic shock to a small number of cultured cells. The lung epithelium cells are interconnected in a vast area structure, which almost uniformly accepts the toxic chemicals per CS inhalation [7,41]. These chemicals are in turn diluted in the existing air volume in the airways so that the resulting toxicity is not instantly detrimental for the epithelium, or the tissues surrounding it. Instead, chronic smoking results in the well-documented loss of the lung internal structures [27], which is due to the accumulation of toxic insults, increased epithelial cell death and a decline in immune cell function.

Exposure of cells to CS by means of CSC or CSE does not provide a reliable simulation system of normal smoking. In human lungs, the inhaled tobacco smoke is extensively diluted (approximately 15 times) due to the huge volume of air inhaled (500 c.c.) after each puff [41]. This dilution of the CS prevents the acute accumulation of a toxic critical mass and the ensuing cell damage, which more than likely happens when either CSC or CSE is used to challenge cultured cells. Furthermore, using either of these methods, it is very difficult to determine the quantity and the quality of the supplied dose and its toxicity. To our knowledge, there has never been in the literature a systematic and quantitative analysis of the tobacco components present in such a preparation. Therefore, it is plausible that only the water-soluble components of CS and a small part of the particulate matter contribute to the toxicity of these preparations. According to our method, the toxic substances in the gaseous phase of CS that are supplied to cells are diluted in a measured air volume within the volumetric chamber so that their contact with the cells simulates normal smoking conditions. In addition, each dose of the GPS supplied has previously been tested for its toxic component load using a well-established method [23].

Previous research mainly focused on the effect of CS on airway epithelium cells, since they are the first cell lineage directly exposed to the toxic effects of tobacco smoke [22,34,35]. Smoking, however, triggers inflammation of the airways, which is brought about by a cascade of events attributed to both innate and acquired immune reactions [27,29]. It is therefore of interest to study the immediate effect of CS on immune cells, as they have the ability to both initiate and perpetuate inflammatory responses in the diseased lung. To date, there are several accounts in the literature that report impaired responsiveness of immune cells -mainly T cells- in murine models that have been attributed to mainstream tobacco smoke [42,43]. It is possible that further research into the impact of CS on immune cells will open up new routes for COPD diagnosis and improve our understanding on the inflammatory versus immunosuppressant cascades, which will allow the design of more effective treatments for the related diseases.

## Conclusion

In conclusion, from our work it is evident that: 1) exposure of cells to low toxicity GPS leads to apoptosis, while high toxicity GPS results in necrotic cell death; 2) supply of GPS should be carried out in an appropriately designed volumetric space, where CS is diluted at approximately the same proportion as in the smoker's airways, and 3) with each supply of CS to cell cultures, prior measurement of the toxic components of CS is necessary, using a well-established and reproducible method.

## Competing interests

The authors declare that they have no competing interests.

## Authors' contributions

AS, ALP and NDS were involved in the conception and experimental design of this study. NDS carried out the western blot analysis and FACS analysis for Annexin V/PI staining and JC-1 monitoring of Δψ_m _depolarization. ALP performed the LDH cytotoxicity assay, the immunofluorescence experiments and the DNA fragmentation analysis. ADV and LHM were responsible for the confocal microscopy images. GB was involved in the flow cytometry analysis. DGH realized the gas chromatography analyses for the obtained GPS samples. All authors read and approved the final manuscript.

## References

[B1] Schumacher JN Green CR Best FW Newell MP Smoke composition: an extensive investigation of the water soluble portion of cigarette smoke J Argic Food Chem 1997 25 310 320 10.1021/jf60210a003 838966

[B2] Barr J Sharma CS Sarkar S Wise K Dong L Periyakaruppan A Ramesh GT Nicotine induces oxidative stress and activates nuclear transcription factor kappa B in rat mesencephalic cells Mol Cell Biochem 2007 297 93 9 10.1007/s11010-006-9333-1 17021677 PMC2758082

[B3] Hogg JC Chu F Utokaparch S Woods R Elliot M Buzatu L Cherniak RM Rogers RM Sciurba FC Coxson HO Pare PD The nature of small-airway obstruction in chronic obstructive pulmonary disease N Engl J Med 2004 350 2645 2653 10.1056/NEJMoa032158 15215480

[B4] Kim V Rogers TJ Criner GJ New concepts in the pathobiology of chronic obstructive pulmonary disease Proc Am Thorac Soc 2008 5 478 485 10.1513/pats.200802-014ET 18453359 PMC2645323

[B5] Churg A Tai H Coultard T Wang R Wright JL Cigarette smoke drives small airway remodelling by induction of growth factors in the airway wall Am J Respir Crit Care Med 2006 174 1327 1334 10.1164/rccm.200605-585OC 17008639

[B6] Ning W Li CJ Kaminski N Feghali-Bostwick CA Alber SM Di YP Otterbein SL Song R Hayashi S Zhou Z Pinsky DJ Watkins SC Pilewski JM Sciurba FC Peters DG Hogg JC Choi AMK Comprehensive gene expression profiles reveal pathways related to the pathogenesis of chronic obstructive pulmonary disease Proc Natl Acad Sci USA 2004 101 14895 14900 10.1073/pnas.0401168101 15469929 PMC522001

[B7] Stavrides JC Lung carcinogenesis: pivotal role of metals in tobacco smoke Free Radic Biol Med 2006 41 1017 30 10.1016/j.freeradbiomed.2006.06.024 16962926

[B8] Yoshie Y Ohshima H Synergistic induction of DNA strand breakage by cigarette tar and nitric oxide Carcinogenesis 1997 18 1359 1363 10.1093/carcin/18.7.1359 9230280

[B9] Yamaguchi Y Nasu F Harada A Kunimoto M Oxidants in the gas phase of cigarette smoke pass through the lung alveolar wall and raise systemic oxidative stress J Pharmacol Sci 2007 103 275 282 10.1254/jphs.FP0061055 17332694

[B10] Stavrides JC Oxidation: The cornerstone of carcinogenesis 2008 Berlin: Springer Science and Business Media B.V;

[B11] Pouli AE Hatzinikolaou DG Piperi C Stavridou A Psallidopoulos MC Stavrides JC The cytotoxic effect of volatile organic compounds of the gas phase of cigarette smoke on lung epithelial cells Free Radic Biol Med 2003 34 345 55 10.1016/S0891-5849(02)01289-3 12543250

[B12] Giordano RJ Lahdenrate J Zhen L Chukwueke U Petrache I Langley RR Fidler IJ Pasqualini R Tuder RM Arap W Targeted induction of lung endothelial cell apoptosis causes emphysema-like changes in the mouse J Biol Chem 2008 283 43 29447 60 10.1074/jbc.M804595200 18718906 PMC2570855

[B13] Betsuyaku T Hamamura I Hata J Takahashi H Mitsuhashi H Adair-Kirk TL Senior RM Nishimura M Bronchiolar chemokine expression is different after single versus repeated cigarette smoke exposure Respir Res 2008 9 7 18 10.1186/1465-9921-9-7 18208591 PMC2248575

[B14] Hasnis E Bar-Shai M Burbea Z Reznick AZ Cigarette smoke-induced NF-kappaB activation in human lymphocytes: the effect of low and high exposure to gas phase of cigarette smoke J Physiol Pharmacol 2007 58 Suppl 5 263 274 18204136

[B15] Yang Q Hergenhahn M Weninger A Bartsch H Cigarette smoke induces direct DNA damage in the human B-lymhoid cell line Raji Carcinogenesis 1999 20 1769 1775 10.1093/carcin/20.9.1769 10469623

[B16] Liu X Togo S Al-Mugotir M Kim H Fang Q Kobayashi T Wang X Mao L Bitterman P Rennard S NF-kappa B mediates the survival of human bronchial epithelial cells exposed to cigarette smoke extract Respir Res 2008 9 66 76 10.1186/1465-9921-9-66 18811964 PMC2567966

[B17] Fujihara M Nagai N Sussan TE Biswal S Handa JT Chronic cigarette smoke causes oxidative damage and apoptosis to retinal pigmented epithelial cells in mice PLoS ONE 2008 3 e3119 10.1371/journal.pone.0003119 18769672 PMC2518621

[B18] Toorn M Van der Slebos DJ de Bruin HG Leuvenink HG Bakker SJ Gans RO Koeter GH van Oosterhout AJ Kauffman HF Cigarette smoke-induced blockade of the mitochondrial respiratory chain switches lung epithelial cell apoptosis into necrosis Am J Physiol Lung Cell Mol Physiol 2007 292 1211 1218 10.1152/ajplung.00291.2006 17209140

[B19] Liu X STAT3 activation inhibits human bronchial epithelial cell apoptosis in response to cigarette smoke exposure Biochem Biophys Res Commun 2007 353 121 126 10.1016/j.bbrc.2006.11.147 17173857

[B20] Liu X Conner H Kobayashi T Kim H Wen F Abe S Fang Q Wang X Hashimoto M Bitterman P Rennard SI Cigarette Smoke Extract Induces DNA damage but not apoptosis in human bronchial epithelial cells Am J Respir Cell Mol Biol 2005 33 121 129 10.1165/rcmb.2003-0341OC 15845867

[B21] Alexandrov K Rojas M Ronaldo C DNA damage by benzo(a)pyrene in human cells is increased by cigarette smoke and decreased by a filter containing rosemary extract, which lowers free radicals Cancer Res 2006 66 11938 11945 10.1158/0008-5472.CAN-06-3277 17178892

[B22] Carnevali S Petruzelli S Longoni B Vanacore R Barale R Cipollini M Scatena F Celi A Giutini C Cigarette smoke extract induces oxidative stress and apoptosis in human lung fibroblasts Am J Physiol Lung Cell Mol Physiol 2003 284 L955 963 12547733 10.1152/ajplung.00466.2001

[B23] Hatzinikolaou DG Lagesson V Stavridou AJ Pouli AE Lagesson-Andrasko L Stavrides JC Analysis of the gas phase of cigarette smoke by gas chromatography coupled with UV-diode array detection Anal Chem 2006 78 4509 16 10.1021/ac052004y 16808460

[B24] Graber R Losa GA Changes in the activities of signal transduction and transport membrane enzymes in CEM lymphoblastoid cells by glucocorticoid-induced apoptosis Anal Cell Pathol 1995 8 159 175 7786813

[B25] Zhang N Hopkins K He YW C-FLIP protects mature T lymphocytes from TCR-mediated killing J Immunol 2008 181 5368 5373 18832693 10.4049/jimmunol.181.8.5368 PMC2562230

[B26] Szabó I Bock J Grassmé H Soddemann M Wilker B Lang F Zoratti M Gulbins E Mitochondrial potassium channel Kv1.3 mediates Bax-induced apoptosis in lymphocytes Proc Natl Acad Sci USA 2008 105 14861 14866 10.1073/pnas.0804236105 18818304 PMC2567458

[B27] Kim V Rogers TJ Criner GJ New concepts in the pathobiology of chronic obstructive pulmonary disease Proc Am Thor Soc 2008 5 478 485 10.1513/pats.200802-014ET PMC2645323 18453359

[B28] Marchetti P Hirsch T Zamzami M Castedo M Decaudin D Susin SA Massee B Kroemer G Mitochondrial permeability transition triggers lymphocyte apoptosis J Immunol 1996 157 4830 6 8943385

[B29] Saetta M Baraldo S Corbino L Turato G Braccioni F Rea F Cavallesco G Tropeano G Mapp CE Maestrelli P Ciaccia A Fabbri LM CD8+ve cells in the lungs of smokers with chronic obstructive pulmonary disease Am J Respir Crit Care Med 1999 160 711 717 10430750 10.1164/ajrccm.160.2.9812020

[B30] Escargueil-Blanc I Salvayre R Nègre-Salvayre A Necrosis and apoptosis induced by oxidised low density lipoproteins occur through two calcium-dependent pathways in lymphoblastoid cells FASEB J 1994 8 1075 1080 7926374 10.1096/fasebj.8.13.7926374

[B31] Raveendram M Wang J Senthil D Wang J Utama B Shen Y Dudley D Zhang Y Wang XL Endogenous nitric oxide activation protects against cigarette smoking induced apoptosis in endothelial cells FEBS Lett 2005 579 733 740 10.1016/j.febslet.2004.12.052 15670837 PMC1350101

[B32] Wang J Wilcken DE Wang XL Cigarette smoke activates caspase-3 to induce apoptosis of human umbellical venous endothelial cells Mol Genet Metab 2001 72 82 88 10.1006/mgme.2000.3115 11161833

[B33] Leist M Single B Castoldi AM Kühnle S Nicotera P Intracellular adenosine triphosphate (ATP) concentration: a switch in the decision between apoptosis and necrosis J Exp Med 1997 185 1481 1486 10.1084/jem.185.8.1481 9126928 PMC2196283

[B34] Hoshino Y Mio T Nagai S Miki H Ito I Izumi T Cytotoxic effects of cigarette smoke extract on an alveolar type II cell-derived cell line Am J Physiol Lung Cell Mol Physiol 2001 281 L509 516 11435227 10.1152/ajplung.2001.281.2.L509

[B35] Jiao ZX Ao QL Xiong M Cigarette smoke extract inhibits the proliferation of alveolar epithelial cells and induces apoptosis Sheng Li Xue Bao 2006 58 244 254 16786109

[B36] Aoshiba K Tamaoki J Nagai A Acute cigarette smoke exposure induces apoptosis of alveolar macrophages Am J Physiol Lung Cell Mol Physiol 2001 281 L1392 1401 11704535 10.1152/ajplung.2001.281.6.L1392

[B37] Wickenden JA Clarke MC Rossi AG Rahman I Faux SP Donaldson K MacNee W Cigarette smoke prevens apoptosis through inhibition of caspase activation and induces necrosis Am J Respir Cell Mol Biol 2003 29 562 570 10.1165/rcmb.2002-0235OC 12748058

[B38] Ishii T Matsuse T Igarashi H Masuda M Teramoto S Ouchi Y Tobacco smoke reduces viability in human lung fibroblasts: protective effect of glutathione S-transferase P1 Am J Physiol Lung Cell Mol Physiol 2001 280 L1189 1195 11350797 10.1152/ajplung.2001.280.6.L1189

[B39] Vayssier M Banzet N François D Bellmann K Polla BS Tobacco smoke induces both apoptosis and necrosis in mammalian cells: differential effects of HSP70 Am J Physiol 1998 275 L771 779 9755110 10.1152/ajplung.1998.275.4.L771

[B40] Slebos DJ Ryter SW vn der Toorn M Liu F Guo F Baty CJ Karlsson JM Watkins SC Kim HP Wang X Lee JS Postma DS Kauffman HF Choi AM Mitochondiral localisation and function of heme oxygenase-1 in cigarette smoke-induced cell death Am J Respir Cell Mol Biol 2007 36 409 417 10.1165/rcmb.2006-0214OC 17079780 PMC1899328

[B41] Stavrides JC Stavrides JC Lungs - Lung volumes and capacities Human Physiology 1997 1 Athens, Greece: Paschalides Medical Publications

[B42] Kalra R Singh SP Savage SM Finch GL Sopori ML Effects of cigarette smoke on immune response: chronic exposure to cigarette smoke impairs antigen-mediated signalling in T cells and depletes IP3-sensitive Ca(2+) stores J Pharmacol Exp Ther 2000 293 166 171 10734166

[B43] Thatcher TH Benson RP Phipps RP Sime PJ High-dose but not low-dose mainstream cigarette smoke suppresses allergic airway inflammation by inhibiting cell function Am J Physiol Lung Cell Mol Physiol 2008 295 L412 421 10.1152/ajplung.00392.2007 18567739 PMC2536795

